# A Statewide Case Management, Surveillance, and Outcome Evaluation System for Children with Special Health Care Needs

**DOI:** 10.1155/2013/793936

**Published:** 2013-03-06

**Authors:** Karen A. Monsen, Scott A. Elsbernd, Linda Barnhart, Jacquie Stock, Carla E. Prock, Wendy S. Looman, Maria Nardella

**Affiliations:** ^1^University of Minnesota, Minneapolis, MN 55455, USA; ^2^Children's Hospitals and Clinics of Minnesota, Minneapolis-Saint Paul, MN 55404, USA; ^3^Children with Special Health Care Needs Program, Washington State Department of Health, Olympia, WA 98504, USA; ^4^Benton-Franklin Health District, Kennewick, WA 98336, USA

## Abstract

*Objectives*. To evaluate the feasibility of implementing a statewide children with special health care needs (CSHCN) program evaluation, case management, and surveillance system using a standardized instrument and protocol that operationalized the United States Health and Human Services CSHCN National Performance Measures. *Methods*. Public health nurses in local public health agencies in Washington State jointly developed and implemented the standardized system. The instrument was the Omaha System. Descriptive statistics were used for the analysis of standardized data. *Results*. From the sample of CSHCN visit reports (*n* = 127), 314 problems and 853 interventions were documented. The most common problem identified was growth and development followed by health care supervision, communication with community resources, caretaking/parenting, income, neglect, and abuse. The most common intervention category was surveillance (60%), followed by case management (24%) and teaching, guidance, and counseling (16%). On average, there were 2.7 interventions per problem and 6.7 interventions per visit. *Conclusions*. This study demonstrates the feasibility of an approach for statewide CSHCN program evaluation, case management, and surveillance system. Knowledge, behavior, and status ratings suggest that there are critical unmet needs in the Washington State CSHCN population for six major problems.

## 1. Introduction 

Children with special health care needs (CSHCN) are at increased risk for poor health outcomes [1]. The CSHCN population is growing, the need for services is increasing, and the capacity to provide CSHCN services is decreasing due to public sector financial constraints [2]. It is critical to demonstrate the needs of this vulnerable population and evaluate the effectiveness and value of CSHCN programs [2, 3]. Public health nurses (PHNs) in Washington State serving CSHCN sought to describe CSHCN client needs and evaluate CSHCN programs using a standardized terminology, the Omaha System [4]. They selected the Omaha System because many of the local Washington State public health jurisdictions used electronic health records and the Omaha System for clinical documentation. Standardized terminologies have potential to advance the development of practice standards and assessment guidelines and overall quality improvement policies and procedures [5–7]. This work builds on previous efforts to describe care and evaluate outcomes in other states and programs [8].

The Maternal Child Health Bureau (MCHB) defines CSHCN as “those who have or are at increased risk for a chronic physical, developmental, behavioral, or emotional condition and who also require health and related services of a type or amount beyond that required by children generally” [1]. The MCHB has advanced a national agenda for achieving and measuring the success of CSHCN programs. The purpose of this agenda is “to provide and promote family-centered, community-based, coordinated care for CSHCN and to facilitate the development of community-based systems of services for such children and their families” [1]. Since the 1980s, this agenda has guided state and federal programs through the identification of six key indicators of progress. These indicators describe the necessity for the early identification of problems in order to provide an opportunity for intervention, as well as the organization of services in order to provide accessible and appropriate interventions [1].

 Identifying health care problems experienced by CSHCN and their families provides information about the larger service system, while a successful system of services results in high levels of child and family health and well-being [9]. Partnering across jurisdictions to define, measure, and monitor a system of care for CSHCN at the state level may lead to the promotion of best practice [10]. Uniform data, specifically, state-level data from the National Surveys of CSHCN, is a key in driving system of care improvements and facilitating state and local program planning efforts [11, 12]. However, little research exists describing the role or benefits of a standardized process for PHN agencies to assess and document nursing activities related to CSHCN programs [13]. 

 Programs that address the unique needs of CSCHN have long been a component of the public health system [2]. Public health nursing contributes a unique service for this population and the health system by assessing health status and access to other health services, assuring that families receive the services they need, and providing findings to policy makers [2]. CSHCN often require long-term services for complex needs, consuming a disproportionate share of health care dollars spent on children. Therefore, they are especially vulnerable to health care issues such as access, quality, and cost containment (denial of care). In Washington State, the CSHCN program goal is to “assure children and youth with special health care needs achieve the healthiest life possible by promoting access to integrated, family-centered, culturally competent, and community-based programs and services” [14].

The adoption of electronic health records and public health information systems varies across jurisdictions in the United States [12]. Many local public health jurisdictions in Washington State implemented commercial and agency-developed public health information systems over the past several years [11]. However, some local public health jurisdictions continue to use paper systems for all data tracking and documentation. The diversity of these documentation methods presents a challenge in uniform statewide data collection. The PHNs developed a solution to this challenge, through the use of a common standardized terminology in all paper or computerized platforms (the Omaha System) [4, 15]. The resulting uniform data could then be combined across all jurisdictions, regardless of how the data were collected.

The objective of this study was to evaluate the feasibility of implementing a state-wide case management, surveillance, and program evaluation system for CSHCN program using a standardized Omaha System protocol and visit report. This paper reports preliminary data from the first four months after the implementation of the standardized data collection protocol.

## 2. Materials and Methods

The University of Minnesota Institutional Review Board and State of Washington Institutional Review Board approvals were obtained for this study. All local public health agencies in Washington State were invited to a statewide training on the data collection system (September 2010) and to participate in the data collection starting November 2010. The data collection period for this study ended in February 2011. Visit reports were submitted to State of Washington Department of Health CSHCN officials for program evaluations by 25 of the 35 local agencies during this time period. The Department of Health entered the deidentified data into a spreadsheet and provided the spreadsheet to the research team for analysis [15].

### 2.1. Instrument: The Omaha System

 The Omaha System [4] was selected by Washington State CSHCN directors for this statewide program evaluation, because it is a standardized interface terminology used widely for computerized documentation in community care settings [4]. It is recognized by the American Nurses Association and other informatics organizations [16]. The Omaha System does not include medical diagnoses or tests. Instead, it provides a comprehensive, holistic architecture for describing, documenting, and evaluating health care by enabling practitioners to collect relevant clinical data and identify client strengths and needs. A previous study compared Omaha System KBS ratings for high risk families including families of CSHCN served in PHN home visiting programs by four local public health agencies in another state (Minnesota) [8]. The Omaha System consists of three components: the Problem Classification Scheme, the Intervention Scheme, and the Problem Rating Scale for Outcomes [4].

The Problem Classification Scheme is used to identify and classify health-related issues and includes 42 problems. Problems are uniquely identified by distinctive definitions and signs and symptoms (s/sx). The Intervention Scheme is used for addressing the problems which are described using the four-level Intervention Scheme. The four terms of the Intervention scheme are: problem from the Problem Classification Scheme, category (action term), target (defined term that further specifies the intervention), and care description (undefined, customizable term) [4].

The Problem Rating Scale for Outcomes is used to measure client knowledge, behavior, and status (KBS) related to each client problem. KBS ratings are documented using a Likert-type ordinal scale from 1 (lowest) to 5 (highest). The knowledge scale measures what the client understands and knows. The behavior scale measures the appropriateness of client actions. The status scale describes the level of severity of client sign and symptoms [4]. The definitions of KBS ratings are provided in Table 1.

### 2.2. Development of the Evaluation Protocol

**Figure 1:**
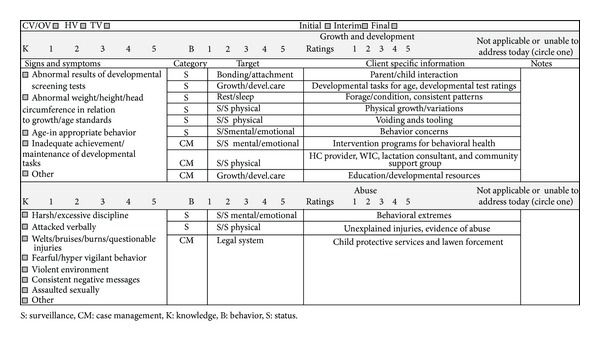
Children with special health care needs visit report example [17].

The State of Washington Department of Health together with PHNs serving CSHCN sought to develop a uniform CSHCN evaluation protocol including the development of practice standards and assessment guidelines. The purpose of their project was to generate data for overall quality improvement and outcomes reporting. They created the evaluation protocol in response to three important trends: (1) the need to respond to federal maternal-child health national performance measures, (2) the fact that many of the public health agencies had the electronic capacity to collect standardized data, and (3) many of the agencies were using the Omaha System for other maternal-child health programs [15]. Title V technical assistance funds supported consultation for this project from a nationally known expert in the use of the Omaha System for program evaluation. PHNs serving CSHCN in Washington State created a logic model for program evaluation based on HHS Title V National Performance Measures (NPM) for addressing CSHCN National Performance Measures (Table 2) [1]. During a ten-month period, PHNs in local public health agencies jointly developed and implemented a standardized visit report operationalizing the National Performance Measures using the Omaha System. Over a series of four meetings, they selected eight problems for the standardized visit report: income, residence, communication with community resources, caretaking/parenting, abuse, neglect, growth and development, and health care supervision. The visit report was available in electronic and paper form (see example in Figure 1). It is available online [17]. Data quality for new Omaha System users was supported by peer training, and by the use of the KBS Rating Guide Supplement, also available online [17].

### 2.3. Sample

 There were CSHCN visit reports (*n* = 127). The client demographics were excluded from analysis because of Washington State data deidentification protocols. The only known characteristics of the sample were that the CSHCN who received PHN visits qualified for services through the HHS Title V CSHCN definitions and criteria.

### 2.4. Variables

 Omaha System variables were problems; s/sx; intervention categories and targets; and knowledge, behavior, and status ratings for client problems. For dependent clients such as CSHCN, the knowledge rating reflects the caregiver's knowledge while behavior and status ratings reflect the child's behavior and status.

### 2.5. Analysis

The Omaha System Partnership for Knowledge Discovery and Health Care Quality [18] provided in-kind data management and analysis for the project. Descriptive statistics were used to quantify the number of clients and s/sx by problem for all problems with 10 or more instances in the data.

## 3. Results

**Figure 2:**
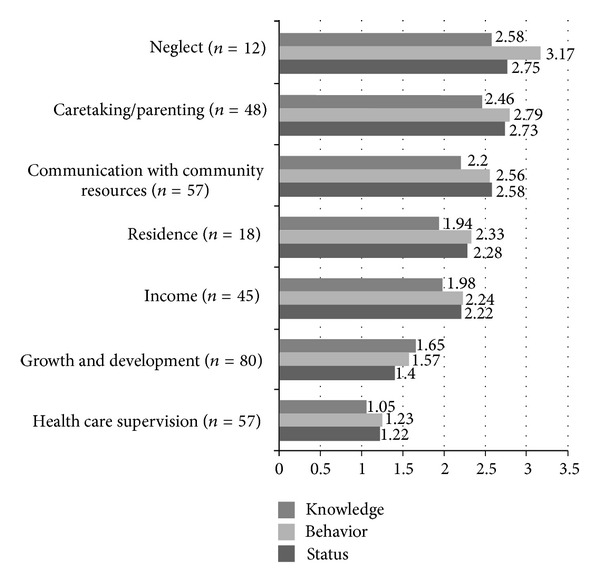
Baseline knowledge, behavior, and status scores for problems of children with special health care needs (*n* = 127).

 From the visit reports (*n* = 127), 314 problems and 853 interventions were documented. The most common problem identified was growth and development followed by health care supervision, communication with community resources, caretaking/parenting, income, neglect, and abuse. The most common intervention category was surveillance (60%), followed by case management (24%) and teaching, guidance, and counseling (16%). On average, there were 2.7 interventions per problem and 6.7 interventions per visit. The mean KBS ratings for each problem are reported in Figure 2.

### 3.1. Growth and Development

 For the growth and development problem (*n* = 80), the most common s/sx were abnormal results of developmental screening tests (40%), inadequate achievement/maintenance of developmental tasks (40%), age-inappropriate behavior (30%), and abnormal weight/height/head circumference in relation to growth/age standards (23%). 

### 3.2. Health Care Supervision

 For the health care supervision problem (*n* = 57), the most common s/sx were inability to coordinate multiple appointments/treatment plans (23%), inadequate source of health care (21%), inadequate treatment plan (21%) fails to obtain routine/preventative health care (18%), inconsistent source of health care (11%), fails to return as requested by health care provider (9%), and fails to seek care for symptoms requiring evaluation/treatment (7%). 

### 3.3. Communication with Community Resources

 For the communication with community resources problem (*n* = 57), the most common s/sx were unfamiliar with options/procedures for obtaining services (58%), language, cultural, educational, transportation barriers (38%), difficulty understanding roles/regulations of service providers (36%), inability to communicate concerns to provider (24%), dissatisfaction with services (20%), limited access to care/services/goods (14%), and inability to use/have inadequate communication devices/equipment (2%). 

### 3.4. Caretaking/Parenting

 For the Caretaking/parenting problem (*n* = 48), the most common s/sx were difficulty providing physical care/safety (33%), difficulty providing cognitive learning experiences and activities (33%), difficulty providing preventative and therapeutic health care (31%), expectations incongruent with the stage of growth and development (19%), difficulty providing emotional nurturance (17%), dissatisfaction/difficulty with responsibilities (15%), difficulty interpreting or responding to verbal/nonverbal communication (15%), and neglectful or abusive (4.2%). 

### 3.5. Income

 The most common s/sx of the income problem (*n* = 45) were low/no income (78%), ability to buy only necessities (33%), uninsured medical expenses (18%), difficulty buying necessities (18%), and difficulty with money management (11%). 

### 3.6. Residence

 For the residence problem (*n* = 18), the most common s/sx were cluttered living space (44%), structural barriers (11%), homelessness (11%), structurally unsound (6%), inadequate heating/cooling (6%), steep, unsafe stairs, (6%) inadequate/obstructed exits and entries (6%), presence of lead based paints (6%), and unsafe equipment/wiring (6%). 

### 3.7. Neglect

For the neglect problem (*n* = 12), the most common s/sx were of the signs and symptoms documented in the neglect problem included *inadequate/delayed medical care *(25%), included *lacks necessary supervision *(17%), while *lacks adequate physical care *(8%)*, lacks emotional support *(8%).

### 3.8. Abuse

Of the 127 visit reports, four abuse problems were recorded, which is less than the threshold of 10 cases established a priori. Therefore, the frequency of s/sx and mean KBS ratings were not reported for the abuse problem. 

## 4. Discussion

The Washington State CSHCN program developed and implemented a state-wide case management, surveillance, and program evaluation system using the Omaha System, demonstrating the feasibility of this approach. Preliminary data suggest serious needs in the CSHCN population that should be further investigated, especially related to health care access for CSHCN and services needed to address developmental issues. The findings of this study were reported to the CSCHN program in April 2011 [15]. The PHNs suggested the very low KBS ratings for the growth and development problem may reflect the fact that funding reductions have severely limited PHN services in local health jurisdictions. As a result, the CSHCN program policy shifted to focus on a small subset of the most seriously affected children. 

### 4.1. Characteristics and Needs of CSHCN and Families

 Omaha System s/sx data provide insight into the characteristics and needs of CSHCN and families. In this study, the s/sx and very low KBS ratings for the health care supervision and communication with community resources problems suggest that there are serious gaps in the sources of health care and treatment plans for CSHCN and that families have difficulty accessing care due to barriers in the system. The s/sx also suggest that very few families are unwilling to access care or use resources. These findings relate to the NPM: “Community resources are organized so families can use them easily,” “Child has a medical home,” and “Families are decision-makers in their child's care and are satisfied with the services they receive.” 

 The s/sx of the caretaking/parenting problem most often related to providing adequate physical care, cognitive learning experiences, and health care. Very few families had s/sx related to parental dissatisfaction or emotional issues. The s/sx of the income problem most often related to income shortages, while few families had budgeting difficulties. These findings relate to the NPM: “Families have adequate public and/or private insurance to pay for the services they need.” 

 The s/sx of the residence problem most often related to excessive household clutter, while few homes had structural deficits and few families were homeless. The most common s/sx of the neglect problem were related to medical neglect and child supervision. Very few CSHCN did not receive appropriate physical care or emotional nurturance. Overall, these results describe the stressful circumstances that families of CSHCN in this study experienced on a daily basis. Consistent with the goal of the CSHCN program, PHNs have traditionally worked with families to promote access to services (program website). The use of the CSHCN visit reports will generate large data sets that will enable further evaluation of the effectiveness of PHN interventions for these problems. Previous studies have described intervention tailoring and effectiveness for high risk families [19–21]. Further research is needed to identify approaches most successful in supporting families of CSHCN, so that resources can be used efficiently and effectively, and the NPM goals for CSHCN and their families can be achieved.

### 4.2. Use of the Omaha System for Uniform Data Collection

 The use of the Omaha System enabled the aggregation of standardized CSHCN data for program evaluation. Assessments and intervention data from 25 of 35 local health jurisdictions were included in this study. The 127 visit reports were submitted to the Department of Health on the paper visit report forms. Data from electronic documentation of CSHCN assessments and interventions were not included in this study but will be available in the future. The interoperability of electronic systems continues to be a challenge. It is essential for state and local health jurisdictions, software vendors, and policy makers to work together to achieve the goals of standardized, interoperable systems to support uniform data collection for program evaluation and research.

### 4.3. Policy Significance

The results of this study will be used by the Washington State CSHCN program to focus nursing activities on the problems most likely to be encountered by PHNs serving the CSHCN population. Additionally, these results will be used to further refine the program evaluation process.

National, state, and local officials have a responsibility to develop, support, and maintain CSHCN programs [1, 22]. Public funding for CSHCN programs in Washington State has been greatly reduced since the inception of this CSHCN program evaluation initiative and continues to be in jeopardy [14, 23]. The data obtained in this study suggest that serious needs are likely to be unmet in the current economic climate due to funding shortfalls and related reductions in CSHCN program staffing. 

### 4.4. Limitations

 With all research using large observational data sets, limitations of the data include observer bias and fidelity to documentation procedures. While the sample of 127 clients from 25 local health jurisdictions demonstrates the feasibility of the approach, bias toward submitting forms for clients with the greatest need may exist. This limitation is supported by the PHN interpretation of the findings reported in Section 5.0. Therefore, alternative explanations for the findings must be considered. In this study, many of the local health jurisdictions started to use the Omaha System for the CSHCN at the beginning of the data collection period. Documentation quality was supported by peer training and the use of KBS rating guide supplements. However, no evaluation of documentation quality was included in the program evaluation protocols. Further research is needed to create quality assurance measures that can be implemented as an integral part of program evaluation.

## 5. Conclusions

This study demonstrates the feasibility of a structured approach to case management, surveillance, and program evaluation for CSHCN using a standardized terminology. The use of the Omaha System facilitated uniform data collection of client assessments and services across 25 of 35 local health jurisdictions in the first four months of the evaluation. Preliminary findings suggest that critical needs existed among CSHCN in Washington State. In the future, larger data sets will be used to evaluate the quality of PHN services, inform public policy, and improve the health CSHCN and their families.

## Figures and Tables

**Table 1:** Definitions of knowledge, behavior, and status ratings [4].

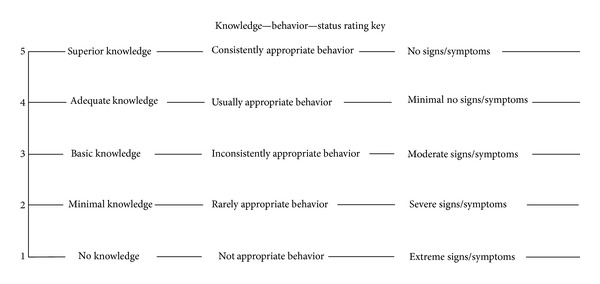

**Table 2:** Proposed outcomes of the project based on national performance measures for children with special health care needs [1] as operationalized by the Omaha System Problem Classification Scheme [4].

NPM: children are screened early and continuously for special health care needs (Growth and development)*	
Developmental screening results are within normal limits or referrals are made	

NPM: child has a medical home (Health care supervision)*	
Caregiver knows when and how to seek emergency, chronic, and acute illness and preventive care	
Caregiver follows prescribed/recommended treatment plan and preventive care	
Consistently uses medical home and health care resources appropriately	
Child receives appropriate timely health care in a medical home	

NPM: community resources are organized so families can use them easily (Communication with community resources)*	
Caregiver is aware of community resources as needed and knows how to access them	
Caregiver uses resources/services consistently	
Caregiver uses resources/services appropriately	

NPM: families have adequate private and/or public insurance to pay for the services they need (Income)*	
Caregiver knows how to navigate resources	
Caregiver completes financial paperwork accurately and on time for medical care	
Health care expenses are covered or paid for	

NPM: families are decision makers in their child's care and are satisfied with the services they receive (Caretaking/parenting)*	
Caregiver knows how to contact CSHCN program as needed.	

NPM: National Performance Measure.

*(Omaha System problems corresponding to each NPM noted in parentheses).
